# Lightweight CNN for accurate brain tumor detection from MRI with limited training data

**DOI:** 10.3389/fmed.2025.1636059

**Published:** 2025-08-29

**Authors:** Awad Bin Naeem, Onur Osman, Shtwai Alsubai, Taner Cevik, Abdelhamid Zaidi, Jawad Rasheed

**Affiliations:** ^1^Department of Computer Science, National College of Business Administration and Economics, Multan, Pakistan; ^2^Department of Biomedical and Neuromotor Sciences, University of Bologna, Bologna, Italy; ^3^Department of Electrical and Electronics Engineering, Istanbul Topkapi University, Istanbul, Türkiye; ^4^Department of Computer Science, College of Computer Engineering and Sciences in Al-Kharj, Prince Sattam Bin Abdulaziz University, Al-Kharj, Saudi Arabia; ^5^Department of Computer Engineering, Istanbul Rumeli University, Istanbul, Türkiye; ^6^Department of Mathematics, College of Science, Qassim University, Buraydah, Saudi Arabia; ^7^Department of Computer Engineering, Istanbul Sabahattin Zaim University, Istanbul, Türkiye; ^8^Department of Software Engineering, Istanbul Nisantasi University, Istanbul, Türkiye; ^9^Research Institute, Istanbul Medipol University, Istanbul, Türkiye; ^10^Applied Science Research Center, Applied Science Private University, Amman, Jordan

**Keywords:** MRI images, deep learning, medical diagnosis, computer-aided diagnosis, healthcare, neuroimaging

## Abstract

**Aim:**

This study aims to develop a robust and lightweight deep learning model for early brain tumor detection using magnetic resonance imaging (MRI), particularly under constraints of limited data availability. Objective: To design a CNN-based diagnostic model that accurately classifies MRI brain scans into tumor-positive and tumor-negative categories with high clinical relevance, despite a small dataset. Methods: A five-layer CNN architecture—comprising three convolutional layers, two pooling layers, and a fully connected dense layer—was implemented using TensorFlow and TFlearn. A dataset of 189 grayscale brain MRI images was used, with balanced classes. The model was trained over 10 epochs and 202 iterations using the Adam optimizer. Evaluation metrics included accuracy, precision, recall, F1 Score, and ROC AUC.

**Results:**

The proposed model achieved 99% accuracy in both training and validation. Key performance metrics, including precision (98.75%), recall (99.20%), F1-score (98.87%), and ROC-AUC (0.99), affirmed the model’s reliability. The loss decreased from 0.412 to near zero. A comparative analysis with a baseline TensorFlow model trained on 1,800 images showed the superior performance of the proposed model.

**Conclusion:**

The results demonstrate that accurate brain tumor detection can be achieved with limited data using a carefully optimized CNN. Future work will expand datasets and integrate explainable AI for enhanced clinical integration.

## 1 Introduction

A technique for training a computer to create original representations from unprocessed data is called deep learning. The network’s popularity may be attributed to its hierarchical and layered structure. Convolutional Neural Networks (CNNs) acquire properties through an object compositional hierarchy, starting with simple edges and progressing to more intricate forms. By layering convolutional and pooling layers, this is achieved. By lowering the feature map, pooling combines similar traits into one, and each convolutional layer identifies local conjunctions of features from the preceding layer. Researchers in neuroscience have also benefited from deep learning, as they are starting to address issues related to neuroimaging. Deep Learning has garnered significant interest due to its ability to address problems across various domains, including medical image analysis. In Palestine, cancer is now the second leading cause of death for both men and women, but over the next decades, it is predicted to overtake all other causes of death (1).

Research has shown that the most effective means of lowering death from brain cancer is early diagnosis and treatment. A low-grade growth that develops slowly will eventually evolve into a neoplasm that grows rapidly. As a result, the first tumor identification and categorization helped to anticipate the prognosis and treatment plan by supporting the assessment of the tumor’s grade and aggressiveness. The diagnosis of brain tumors is mostly reliant on medical imaging (2). One of the most efficient methods currently used for tumor detection is magnetic resonance imaging (MRI). A powerful magnetic flux, radiofrequency pulses, and a laptop is employed to process tomography imaging data to produce detailed images of soft tissues and organs. It aids medical professionals in treating illnesses. The main reason for tomography’s popularity is that it is a more suitable designation than X-rays (3).

Noise significantly degrades medical images, including MRIs. This is largely due to knowledge acquisition systems, multiple sources of interference, operator error, and other factors that impact imaging mensuration processes and can lead to significant classification errors (4). This approach typically requires a basic microscope and may result in a different or incorrect diagnosis, yet it is often inappropriate when dealing with human life. It emphasizes the need for power-assisted systems, high-precision systems, or diagnostic systems (CADx) (5). The CADx system is essential for medical institutions, as it supports the judgments made by doctors and radiologists. It may be challenging to create a highly automated and economical diagnostic system as a result (6).

Gliomas are the most prevalent and aggressive kind of brain tumor, with a very short survival time for the highest grade. Therefore, therapy planning may be a crucial step in raising the medical patients’ standards of living. One popular imaging modality for evaluating these tumors may be MRI (7). These days, with numerous instances and massive volumes of objective data analysis, computer-based medical image analysis is gaining popularity due to its speed and intelligence, surpassing manual methods. By varying the excitation and repetition durations, magnetic resonance imaging may produce notably unique tissue types, making it an incredibly adaptable tool for studying various structures of interest. A single magnetic resonance imaging scan is insufficient to phase the growth and all of its subregions fully. Convolutional Neural Networks (CNNs) have demonstrated high effectiveness in identifying cell division events in two-dimensional microscopic anatomy pictures within the field of medical image analysis. When it comes to machine learning strategies, deep learning is undoubtedly the best option for many imaging tasks. The possibility of deep learning-based automated diagnosis of brain illnesses will arise from the availability of large neuroimaging data sets for training. MRI is a frequently used medical imaging method that offers information on the identification of brain tumors (8). One of the main challenges a physician has after reviewing the tomography data is determining how much time and effort to devote to tumor detection. These days, CNNs are used for the majority of picture classification problems due to their superior accuracy and precision over other currently used techniques. The accuracy and precision of tumor detection and identification have increased due to the use of CNNs for image classification (9).

## 2 Related work

Over the last 20 years, the detection of brain cancers using MRI has undergone significant advancements, thanks to the integration of deep learning (DL), traditional machine learning (ML), and conventional image processing techniques. This section discusses the main categories of methodologies and provides an overview of how our research contributes to and expands upon the existing body of literature.

### 2.1 Conventional techniques for machine learning and segmentation

Most of the early work uses unsupervised clustering and custom feature extraction. Due to their ability to separate picture intensities into clusters that represent normal and diseased tissue regions, segmentation techniques like fuzzy C-Means (FCM) and K-Means clustering have been widely used (10–12). Despite achieving basic localization, these methods were very susceptible to noise and required human parameter adjustment. Changes aimed at improving segmentation accuracy, such as region-expanding algorithms (13, 14) and gray-level histograms (15), were computationally expensive and inconsistent, particularly in low-contrast or early-stage tumors where borders were not obvious. For feature extraction and classification, further research employs learning vector quantization, support vector machines (SVMs), and artificial neural networks (ANNs) (16, 17). These earlier methods, however, sometimes did not work with diverse patient datasets and needed careful feature engineering.

### 2.2 Techniques based on deep learning and CNN

CNNs have been used extensively in medical imaging applications due to their effectiveness in computer vision (26, 27). CNNs eliminate the requirement for human feature design by automatically extracting hierarchical features. Models like AlexNet, VGG16, and ResNet have been modified to perform tasks related to brain tumor classification and segmentation (18, 19). Although these designs have demonstrated outstanding performance, they often rely on large, annotated datasets, which are challenging to collect in the medical field due to privacy concerns and high labeling costs. To manage volumetric MRI data and capture spatial relationships between image slices, 3D CNNs have been the subject of several studies (20). Although these models improve the accuracy of segmentation tasks, their computational cost makes them unsuitable for real-time applications or situations with limited resources. Similar studies have been conducted on Stacked Autoencoders (SAEs) and Deep Belief Networks (DBNs) (21), but in the lack of suitable data, training these deep models from scratch may lead to overfitting.

### 2.3 Domain adaptation and learning transfer

By utilizing pre-trained networks as feature extractors for MRI classification, which have been trained on natural image datasets such as ImageNet, researchers have employed transfer learning to reduce the need for large datasets (22, 23). When paired with domain-specific fine-tuning, it can accelerate training and enhance generalization. However, insufficient feature representations may result from the domain mismatch between natural and medical images. ResNet or InceptionV3 versions that have been carefully altered and work well on binary classification tasks are used in certain studies. Clinical safety criteria, such as recall and AUC, which are essential for real-world diagnosis, are seldom used to evaluate models.

### 2.4 Methods for multimodal MRI and synthesis

To collect different tissue contrasts, advanced segmentation algorithms often use several MRI modalities. Studies like the BraTS Challenge and BraSyn Benchmark (24, 25) demonstrate the challenges that arise when sequences are erratic or nonexistent, while also emphasizing the advantages of multimodal input. To fill in the gaps, several studies have explored the creation of synthetic MRIs using GANs or autoencoders; however, these methods require a complex design and are not ideal for use in situations with limited data.

## 3 Materials and methods

Cancer remains one of the most life-threatening diseases worldwide, and early detection is critical for effective treatment. MRI is a widely used, non-invasive imaging technique that helps identify abnormalities in the brain, including cancerous tumors. In recent years, machine learning—particularly image classification techniques—has demonstrated significant promise in improving the accuracy and speed of cancer detection using MRI. This study examines the DL-based application in developing a CNN for brain tumor detection using MRI scans. The proposed CNN architecture consists of five layers, specifically designed to classify MRI images into cancerous and non-cancerous categories with high accuracy.

### 3.1 Data acquisition

Data plays a crucial part in machine learning systems. The dataset utilized in this work was available from the UCI Machine Learning Repository and Kaggle, both of which are publicly accessible. The dataset downloaded from Kaggle and is accessible at https://www.kaggle.com/datasets/navoneel/brain-mri-images-for-brain-tumor-detection/data (last accessed: January 10, 2025), and the second dataset is available at https://www.kaggle.com/datasets/sartajbhuvaji/brain-tumor-classification-mri (last accessed: January 20, 2025).

### 3.2 Methodology and model architecture

The architecture employed in this study is based on a CNN design, which is particularly effective for image classification tasks. CNNs typically include the following core components:

- Convolutional Layers: Extract feature maps from the input image using learned filters and apply non-linear activation functions (e.g., ReLU).
- Pooling Layers: Reduce the spatial size of feature maps, enhance computational efficiency, and mitigate overfitting—max-pooling is the most commonly used technique.
- Fully Connected (Dense) Layers: Interpret the extracted features and produce classification decisions; each neuron is connected to all neurons in the previous layer.

The proposed model consists of five primary layers: three convolutional layers, two max-pooling layers, and a fully connected dense layer. The architecture is implemented using the high-level TensorFlow Layers API, which streamlines the creation of neural networks by offering functions to define convolutional, pooling, and dense layers, along with activation functions and regularization options such as dropout.

**Figure 1:**
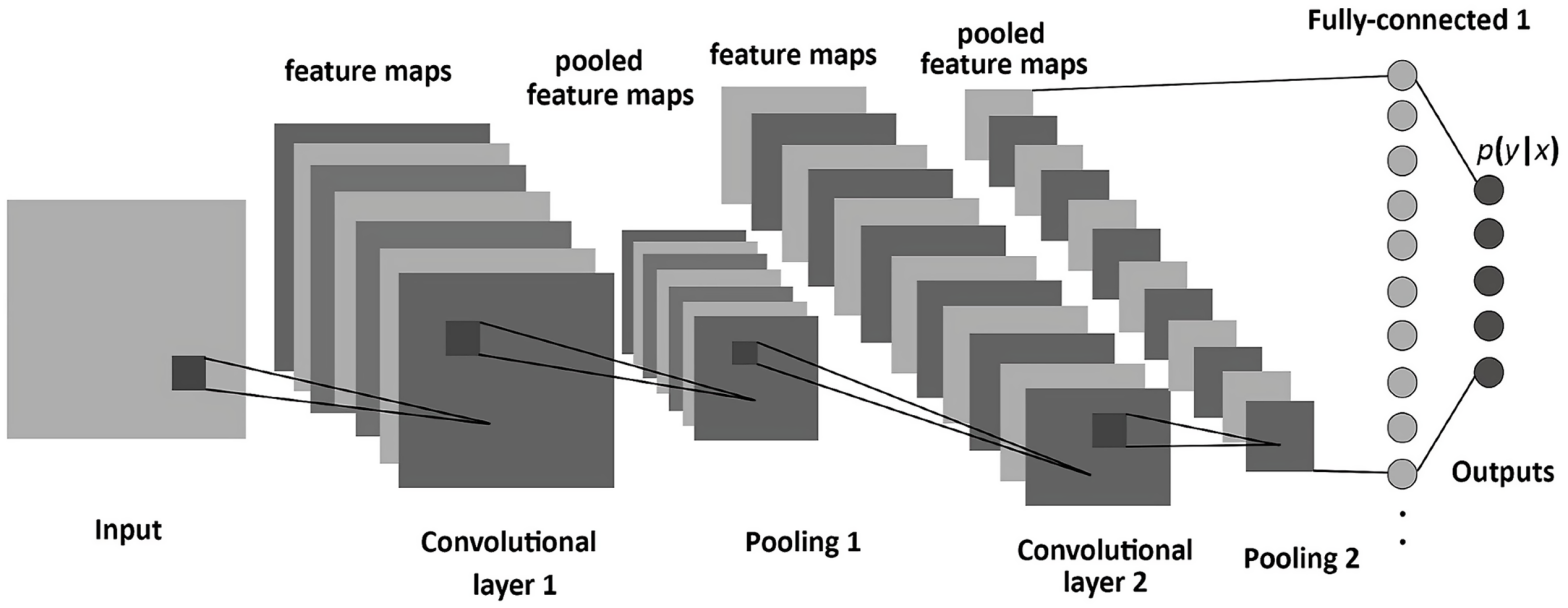
CNN architecture for brain tumor classification, showing layers for feature extraction and final classification from MRI input images.

Figure 1 illustrates the sequential layer-wise architecture of the CNN, clarifying the dimensional transformation of MRI data from input through convolution, pooling, and dense layers to the final binary classification. The model was trained using the Adam optimizer with the following parameters: *ε* = 1e-8, *β*₁ = 0.9, *β*₂ = 0.999, and a learning rate of 0.001. To avoid overfitting, a dropout layer with a 0.5 rate was added after the dense layer.

The model processes grayscale MRI images resized to 128 × 128 × 1. The first convolutional layer applies 32 filters (3 × 3) with ReLU activation, followed by a 2 × 2 max-pooling operation. The second convolutional layer utilizes 64 filters (3 × 3) with ReLU activation and an additional 2 × 2 max-pooling operation. The third convolutional layer consists of 128 filters (3 × 3), followed by another pooling operation. The output of the convolutional stages is flattened and passed to a dense layer with 128 neurons, also using ReLU activation. Ultimately, a single output neuron with sigmoid activation yields a binary classification decision (tumor-positive or tumor-negative).

**Figure 2:**
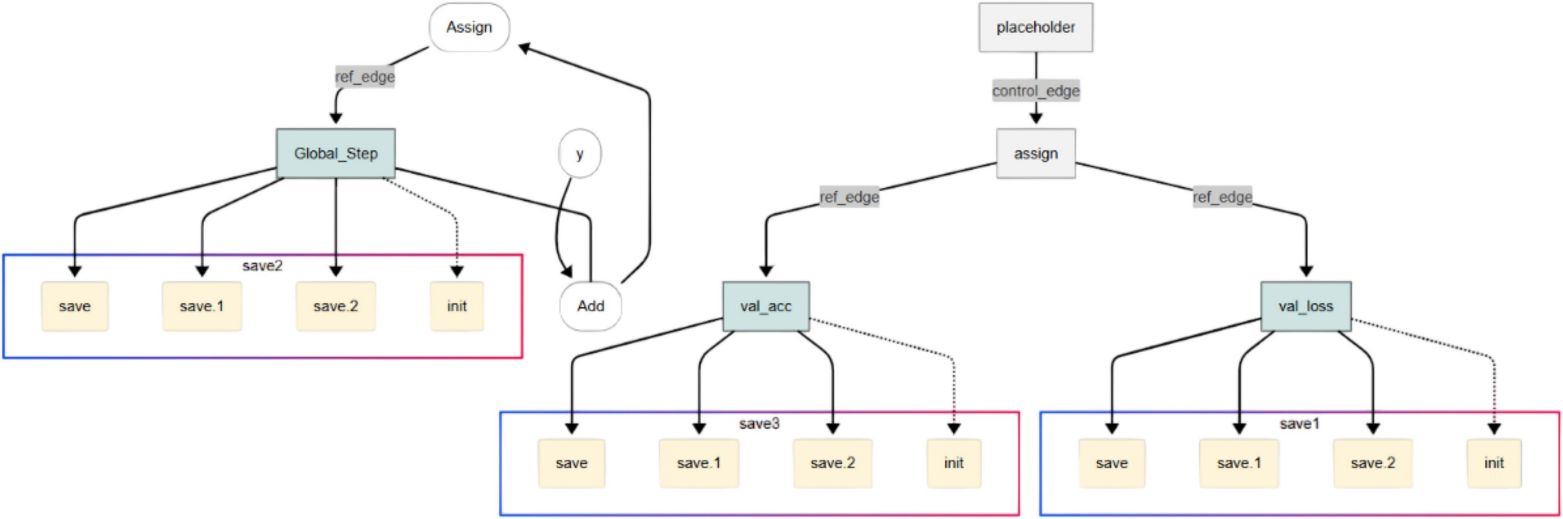
Feature extraction in early CNN layers showing low-level spatial features such as edges and textures derived from tumor MRI images.

Figure 2 illustrates the initial layers of the CNN, including the first convolution and pooling layers. The initial convolutional and pooling layers extract low-level spatial features, such as edges and texture gradients, which are essential for differentiating tumor boundaries from normal tissue in MRI images, including edges, lines, and simple textures. The visual representation highlights how spatial information is preserved while dimensionality is reduced.

**Figure 3:**
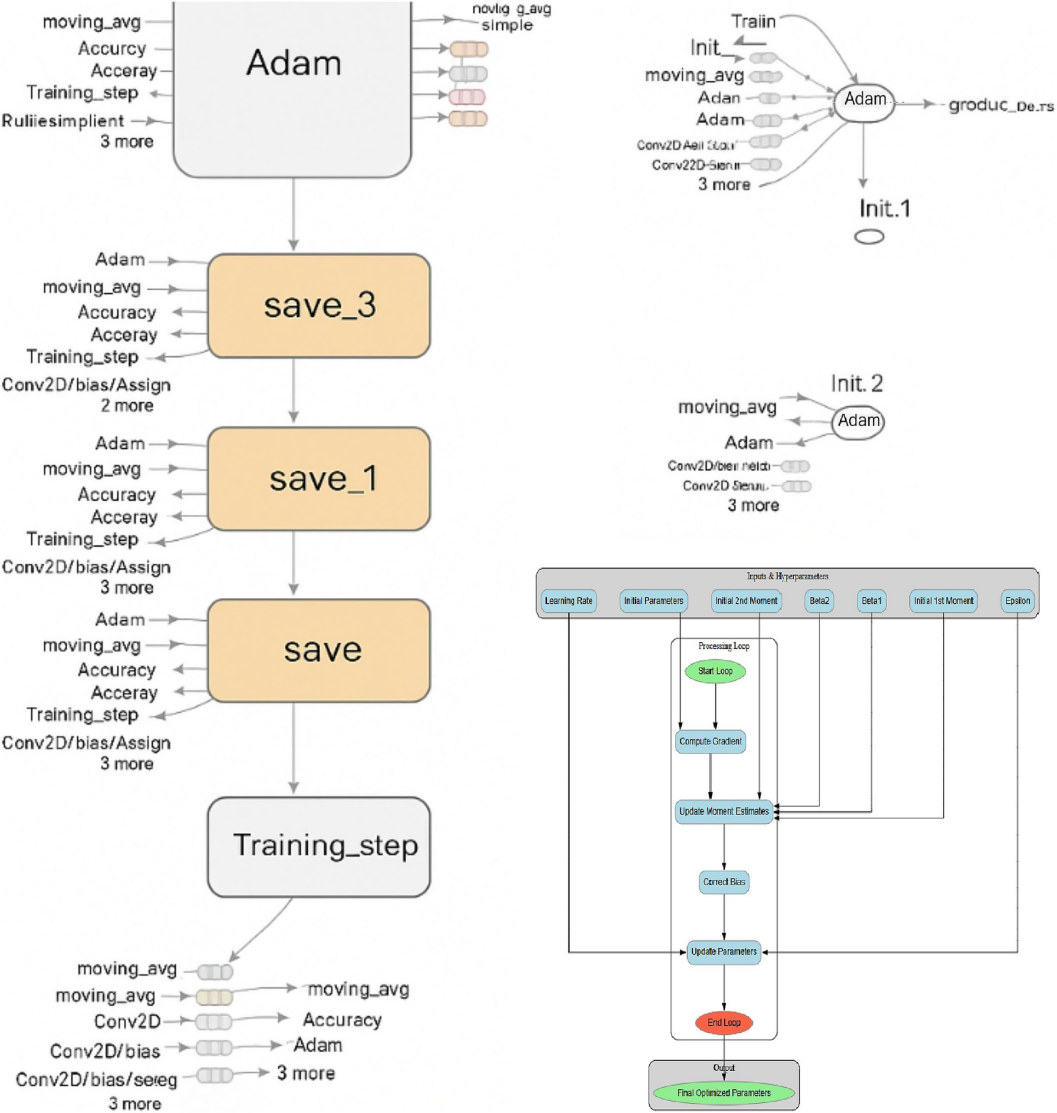
Intermediate CNN layers highlighting deeper convolutions and expanded feature maps that capture high-level tumor features.

Figure 3 illustrates the intermediate layers of the CNN, which include deeper convolutional layers with a greater number of filters. These layers extract high-level, abstract features such as tumor shapes, boundaries, and textures. These deeper layers abstract high-level semantic features such as irregular tumor shapes, enhancing the model’s ability to distinguish pathological from healthy brain structures.

**Figure 4:**
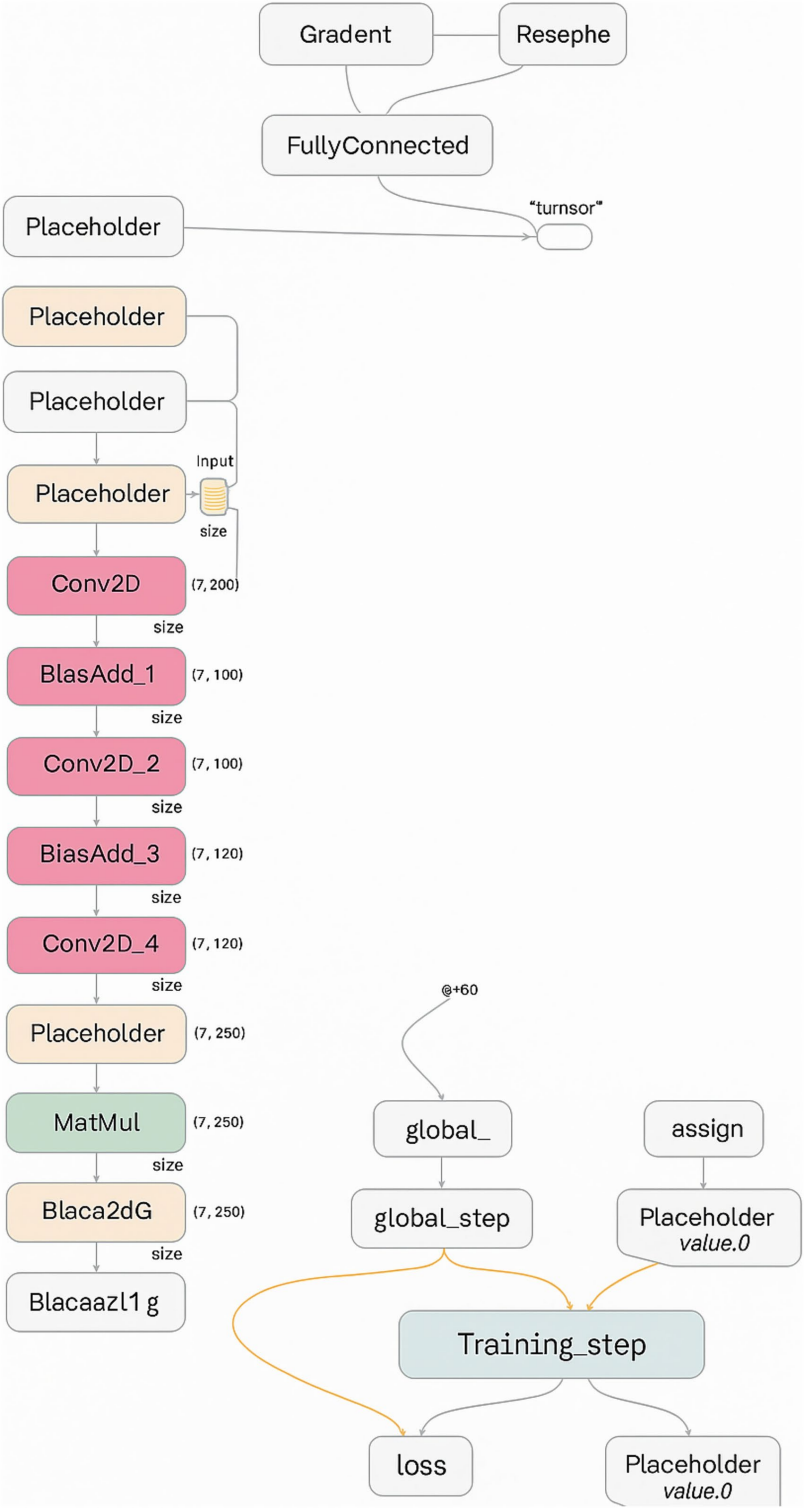
Final CNN layers: dense and sigmoid output units responsible for probabilistic classification of tumor presence.

Figure 4 focuses on the final layers of the CNN, including the fully connected dense layer and the output neuron. These layers are responsible for interpreting the extracted features and making the final classification decision. The use of sigmoid activation in the output neuron enables the model to output a probability score indicating the presence or absence of a brain tumor.

To complement these visual representations, Table 1 provides a detailed layer-wise summary of the CNN model, listing input/output dimensions, number of filters or neurons, kernel and pooling sizes, and activation functions used at each stage. Moreover, it offers a concise yet thorough reference for understanding the architecture’s design and function.

**Table 1:** Layer-wise architecture of the proposed CNN model, detailing input/output shapes, filter counts, kernel sizes, activation functions, and pooling operations for each layer.

Layer type	Output shape	Activation	Notes
Input Layer	(128, 128, 1)	—	Grayscale MRI input
Conv2D	(128, 128, 32)	ReLU	32 filters, 3 × 3 kernel
MaxPooling2D	(64, 64, 32)	—	2 × 2 pool size
Conv2D	(64, 64, 64)	ReLU	64 filters, 3 × 3 kernel
MaxPooling2D	(32, 32, 64)	—	2 × 2 pool size
Conv2D	(32, 32, 128)	ReLU	128 filters, 3 × 3 kernel
MaxPooling2D	(16, 16, 128)	—	2 × 2 pool size
Flatten	(32768)	—	—
Dense	(128)	ReLU	Fully connected layer
Output (Dense)	(1)	Sigmoid	Binary classification output

The TensorFlow Layers API enables the construction of these components with functions such as:

- conv2d(): Defines 2D convolutional layers with specified parameters.
- max_pooling2d(): Creates pooling layers to down-sample feature maps.
- dense(): Builds fully connected layers for classification.

Due to the complexity of the computational graph, it is segmented for clarity across Figures 2–4, with each segment representing a critical stage in the data transformation and classification process.

## 4 Experimental setup and results

The proposed CNN model was trained and evaluated using a dataset comprising 189 MRI images, with an equal balance between cancerous and non-cancerous cases. The dataset was stratified into training, validation, and testing subsets to maintain balanced representation of tumor-positive and tumor-negative cases. Table 2 presents the data distribution according to the train and test splits. Training was performed for 10 epochs with a batch size of 18, yielding approximately 202 iterations. Key performance metrics, including accuracy, loss, and ROC-AUC, were monitored via TensorBoard throughout training. Hyperparameters were consistently maintained across experiments to enhance reproducibility. Tracking accuracy and loss over 202 iterations with TensorBoard enabled validation of stable convergence and early detection of overfitting, which is critical given the limited dataset size.

**Table 2:** Dataset distribution across training, validation, and testing subsets, showing balanced representation of tumor-positive and tumor-negative MRI scans of the first dataset.

Dataset split	Number of images	Tumor-positive	Tumor-negative
Training	133	67	66
Validation	28	14	14
Testing	28	14	14
Total	189	95	94

Because of the small sample size, we utilized TensorFlow’s “ImageDataGenerator” to supplement data in real time and increase generalization. The augmentation pipeline used horizontal flipping (*p* = 0.5) to mimic mirrored brain orientations, small-angle rotations (±10°) to account for head tilt variability, random zoom (±5%) and translations (±5% of image dimensions) to simulate patient positioning differences, and Gaussian noise injection (*σ* = 0.01) to simulate MRI scanner acquisition noise. The augmentation pipeline contained:

- Horizontal Flipping: To represent mirrored anatomical configurations, has a chance of 0.5.
- Rotation: Random small-angle rotations within ±10°, to account for minor patient head tilts.
- Zoom: To mimic size differences across scanners, zoom in and out by up to 5%.
- Translation: An image dimension from vertical and horizontal shift up to 5%.
- Noise injection: MRI scanner acquisition noise is simulated using low-level Gaussian noise (*σ* = 0.01).

To accommodate for changes in intensity from scanner calibration, adjust brightness by ± 10%. To expose the model to a broader variety of real-world input conditions without needlessly extending the dataset on disk, these modifications to the training set were performed stochastically throughout each epoch. Each run started with a predefined random seed to maintain consistency. We can assure repeatability and back up our claims of strong generalization with short datasets by enabling other researchers to reproduce our preprocessing pipeline and see whether analogous augmentation tactics offer equivalent advances in other limited-data settings. In clinical contexts with limited and varied patient data, augmentation decreases overfitting, enhances feature diversity, and makes the model more usable.

The dataset used in this study consisted of MRI scans collected from multiple patients, with one representative scan per subject to minimize redundancy and prevent model bias. In cases where numerous scans were available per patient, only one scan was randomly selected to ensure that no patient’s data appeared in both the training and validation sets. This procedure prevents data leakage, ensuring that the model’s performance reflects genuine generalization rather than memorization of individual patient characteristics.

**Figure 5:**
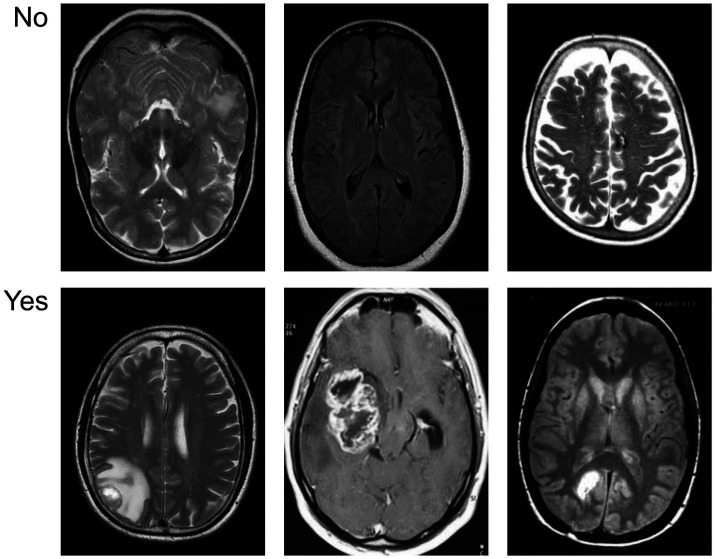
Sample visualization of the MRI dataset illustrating differences between tumor-positive and tumor-negative brain images.

Figure 5 provides a visual overview of the dataset used in our experiments, distinguishing between cancerous and non-cancerous MRI brain scans. Our CNN effectively captured these differences in structural patterns and intensities for classification.

The model was trained for 35 epochs (840 iterations), achieving a peak validation accuracy of 98%. The model’s high precision and recall indicate its potential as a clinical decision support tool to aid radiologists in more efficient brain tumor identification. Each training example that passes through the network in both forward and backward propagation constitutes one iteration.

The Adam optimiser was configured with a learning rate of 0.001, *β*₁ = 0.9, β₂ = 0.999, and *ε* = 10^−8^. These values are known to offer stable and efficient convergence in deep learning models, especially when working with small datasets. They were selected after preliminary tuning and cross-referencing with prior studies demonstrating similar use cases in MRI image classification. Although extensive hyperparameter tuning was beyond the scope of this study, the choice of hyperparameters was based on standard values widely adopted in the literature for medical image classification tasks.

**Figure 6:**
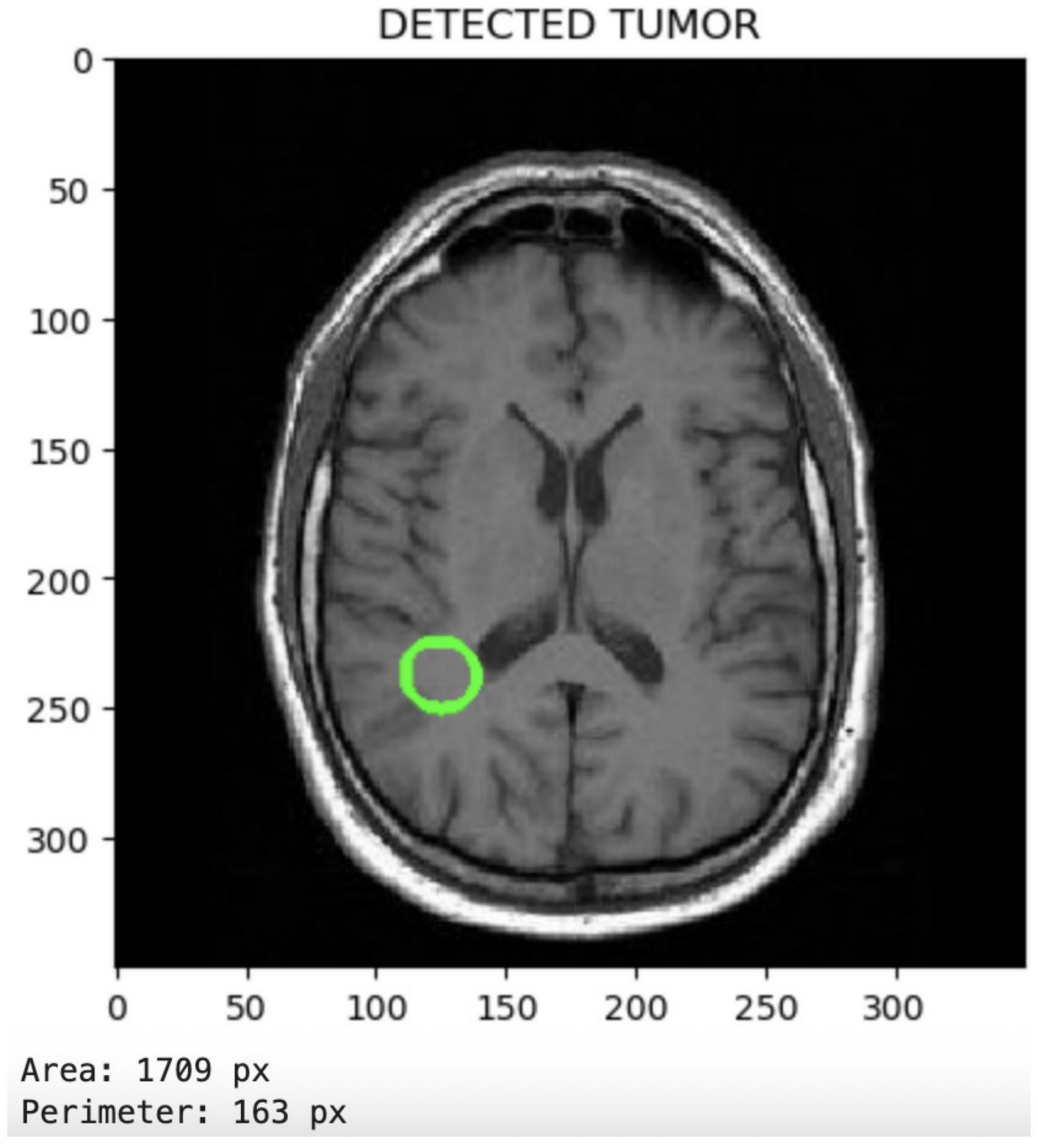
Segmentation output visualizing localized tumor regions, highlighting the model’s spatial discrimination capabilities.

Figure 6 displays the tumor segmentation output, highlighting spatial tumor regions. The trained model not only classifies the presence of tumors but also enables the visualization of the detected tumor region. This segmentation capability adds clinical value by providing spatial context for the tumor’s location and size.

**Figure 7:**
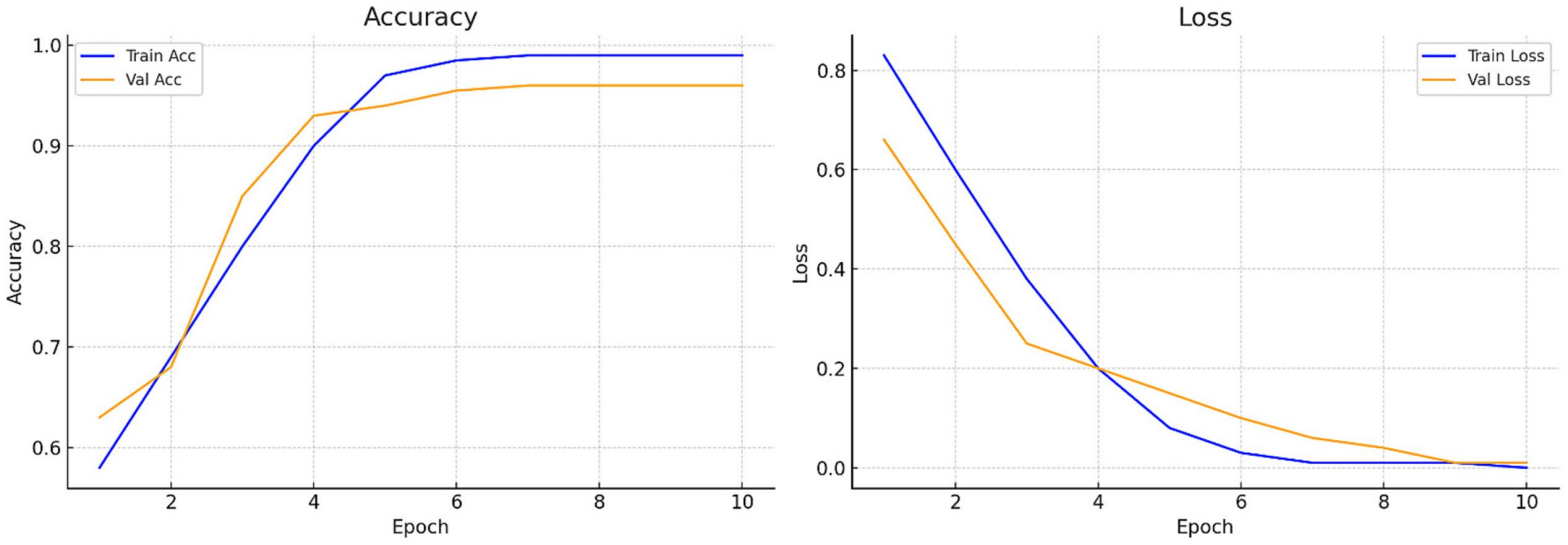
Accuracy and loss curves of the proposed model: Training loss progression illustrating reduction from 0.412 to near zero, reflecting stable model convergence.

**Figure 8:**
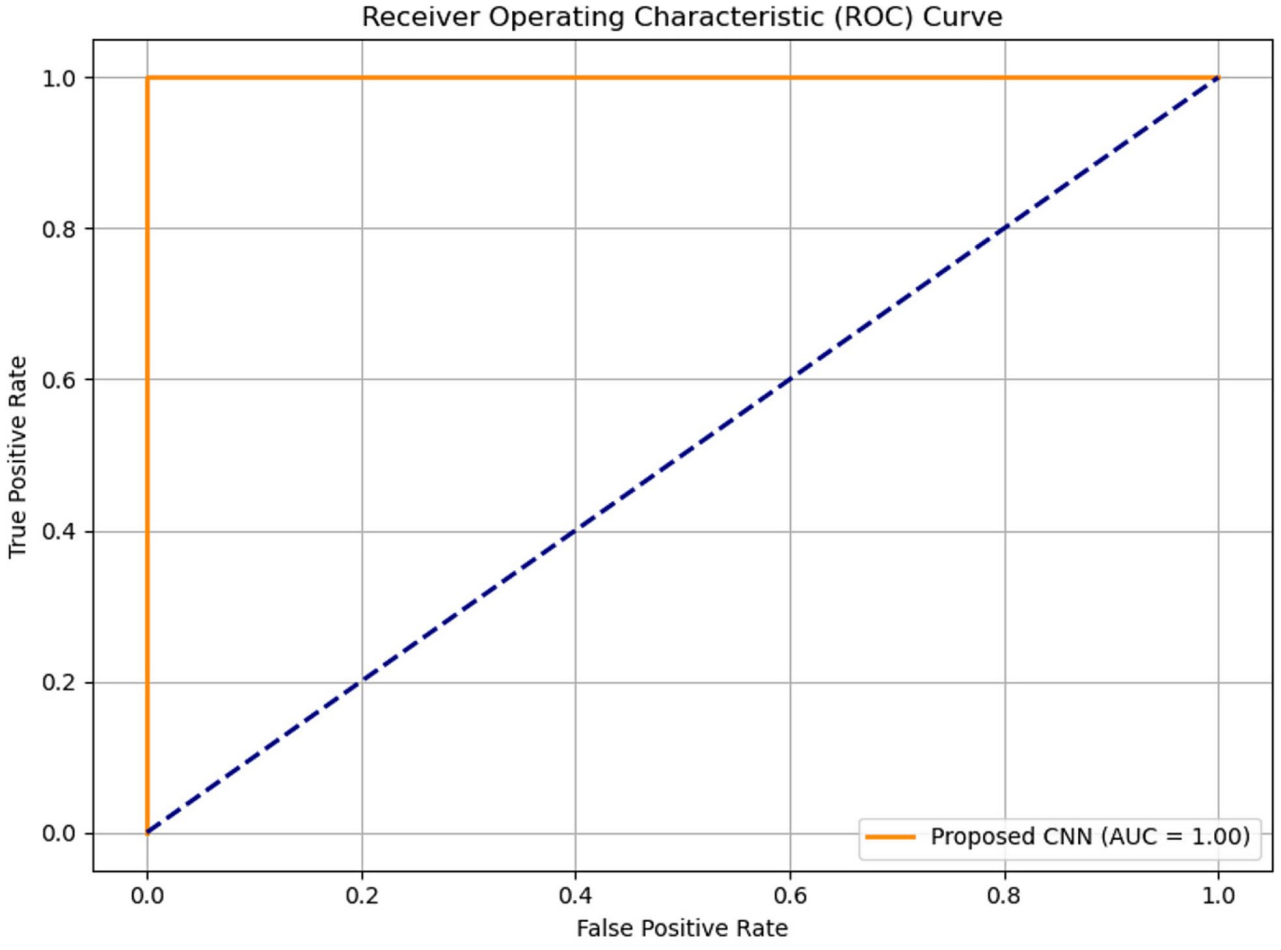
Receiver Operating Characteristic (ROC) curve of the proposed model with an AUC of 0.99, indicating excellent diagnostic accuracy.

Figure 7 illustrates the accuracy across iterations, which initially shows an uneven distribution but ultimately converges to zero as the iterations progress. The loss rate is a critical component of CNN and is used to improve the CNN architecture. Despite the limited dataset, the proposed model effectively minimizes loss and enhances accuracy. Figure 8 presents the Receiver Operating Characteristic (ROC) curve with an AUC of 0.99, illustrating excellent diagnostic ability.

**Figure 9:**
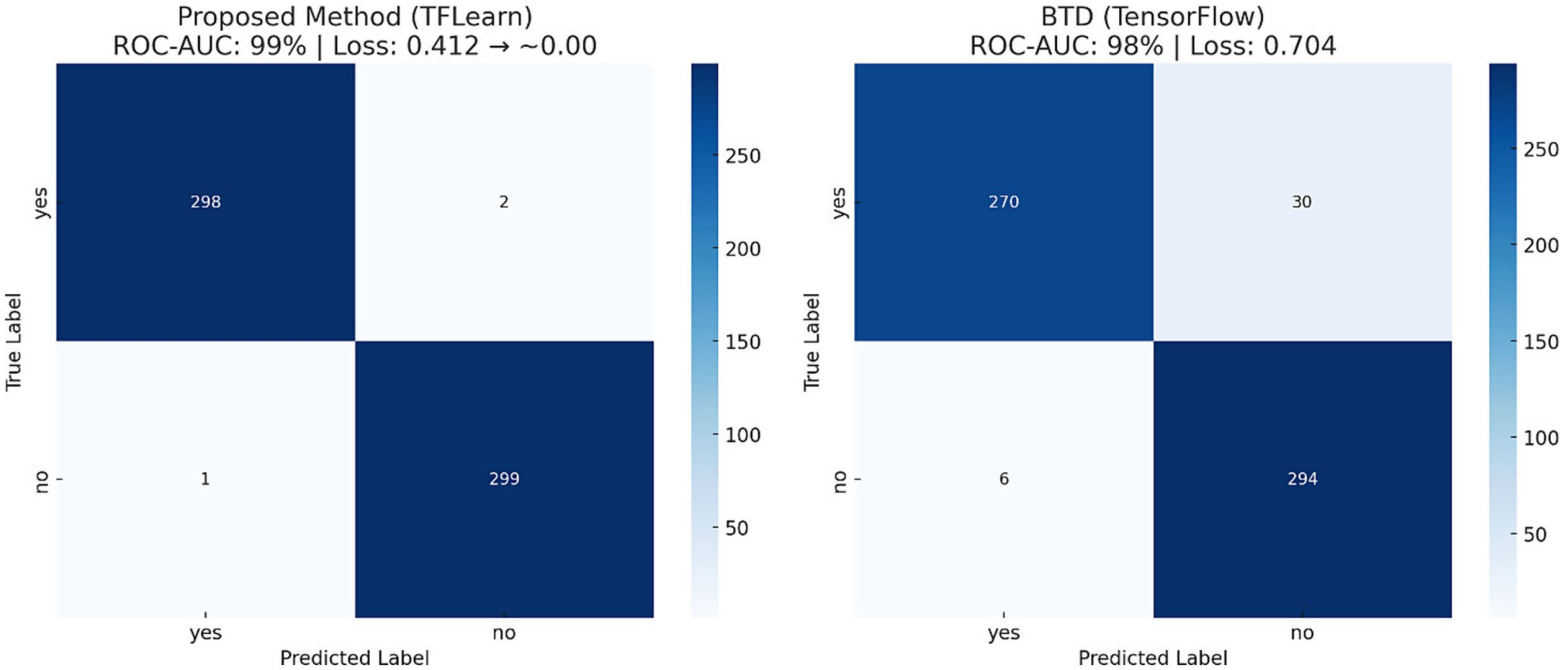
Confusion matrix showing true positive and true negative predictions, validating classification reliability.

To further assess the performance of the proposed CNN-based model, standard classification metrics were computed, including precision, recall, F1-score, accuracy, and the area under the ROC-AUC curve. Table 3 consolidates critical performance metrics, including training accuracy (99%), validation accuracy (99%), loss rate reduction from 0.412 to nearly zero, precision, recall, F1-score, and ROC-AUC (0.99), providing a clear and concise overview of the model’s effectiveness. Figure 9 illustrates the confusion matrix of both proposed and baseline models when tested with 600 test images of the second dataset. Additionally, Table 4 compares the performance of the proposed model with a baseline TensorFlow model trained on a larger dataset (1800 images) that has lower accuracy (98%) and higher loss (0.704). The proposed CNN model has superior performance despite the limited data.

**Table 3:** Performance metrics of the proposed CNN model, including accuracy, precision, recall, F1-score, ROC-AUC, and reduction in loss rate.

Metric	Value
Accuracy	99.00%
Precision	98.75%
Recall	99.20%
F1 Score	98.87%
ROC-AUC	0.99
Loss Reduction	0.412 → ~0.00

**Table 4:** Comparative evaluation of the proposed CNN model versus a baseline TensorFlow implementation, highlighting improved performance with fewer training samples.

Method	Epochs	Iterations	Dataset	ROC-AUC	Loss rate
BTD (TensorFlow)	35	840	1800	98%	0.704
Proposed Method (TFLearn Based)	10	202	189	99%	0.412 → ~0.00

The five-layer CNN architecture was selected to balance classification accuracy and computational efficiency on a limited dataset for prospective clinical use. Early research compared the recommended design to a more complex 8-layer CNN with an extra convolution-pooling block and a second dense layer. Despite reaching 99% training accuracy, the deeper model’s validation accuracy plateaued at 96% after the 20th epoch and displayed peculiar loss oscillations, indicating overfitting due to the limited dataset size of 189 pictures. Across all training and validation sets, the five-layer model consistently reduced loss from 0.412 to near zero while maintaining 99% accuracy, demonstrating strong generalization capabilities. Furthermore, it reduced the number of parameters by approximately 38%, thereby decreasing training time on the same GPU from 7.8 s to 4.9 s per epoch. This efficiency directly supports the study’s purpose of creating a lightweight diagnostic model suited for real-time inference in clinical settings, especially when resources are constrained. The architect’s decision reflects the nature of the classification challenge. When utilizing MRI to identify brain cancers, spatial indicators such as tumor margins, regional intensity variations, and abnormal textural patterns are crucial. They may be successfully retrieved without having a massive network depth by utilizing three progressively deeper convolutional layers (32, 64, and 128 filters). According to feature map representations, the proposed CNN properly captured both low-level edge attributes and higher-level tumor form abstractions that were comparable to those in the deeper model. Given the dataset, processing settings, and observable performance limits, the five-layer CNN delivers the ideal blend of accuracy, resilience, and efficiency for this experiment.

## 5 Conclusion

Deep learning has become a crucial tool in biomedical image analysis, particularly for applications such as brain tumor classification using MRI scans. For quicker model construction, the proposed technique employs CPU-based TensorFlow and TFLearn, as well as GPU-based TensorFlow. Deep learning (DL) techniques are increasingly employed in medical imaging for brain tumor detection and classification. The use of MRI is essential for detecting abnormal brain tissues, and accurate tumor diagnosis is vital for treatment planning. To categorize and diagnose brain tumors from a limited MRI dataset, the study employs a deep learning approach using a Convolutional Neural Network (CNN). The proposed model achieved 99% training and 99% validation accuracy, with a validation loss reduction from 0.412 to near 0.000 across 10 epochs. Additionally, the model attained an ROC-AUC of 0.99, confirming its strong discriminative capability. The proposed CNN model outperformed a baseline model trained on a larger dataset, achieving higher accuracy (99% vs. 98%) and lower validation loss (0.412 vs. 0.704), which indicates strong potential for deployment in real-time clinical diagnostics, especially in data-limited settings. The suggested CNN model may be used in real-world healthcare environments because of its lightweight design and exceptional diagnostic precision. In a radiology department’s existing PACS (Picture Archiving and Communication System), a radiologist may use the model as an automated pre-screening tool to rank MRI images with a high likelihood of tumor incidence. Real-time feedback during diagnostic sessions could be provided by integrating the model with clinical decision support systems. Additionally, report authoring could be made easier by connecting to Radiology Information Systems (RIS). Because of its minimal computational requirements (4.9 s per epoch on a standard GPU), the model may also be implemented on-site in hospitals with limited resources, eliminating the need for cloud-based processing. Regulatory approval, interoperability with different MRI scanner outputs, and further validation across multiple-center datasets to ensure robustness are the remaining challenges. Before clinical utilization is widely accepted, these challenges need to be resolved.

## 6 Future directions

Future work will focus on expanding the dataset to improve model generalization and reduce bias. Integrating additional imaging modalities, such as Computed Tomography (CT) and Positron Emission Tomography (PET), as well as utilizing transfer learning with pre-trained models, may enhance performance. Exploring three-dimensional Convolutional Neural Networks (3D CNNs) can capture spatial context more effectively, while explainable AI methods, such as Gradient-weighted Class Activation Mapping (Grad-CAM), can improve interpretability. In the future, data augmentation techniques, including rotation, flipping, scaling, and brightness adjustment, can be employed to assess the model’s generalization.

## Data Availability

Publicly available datasets were analyzed in this study. This data can be found here: the datasets analyzed for this study can be found at https://www.kaggle.com/datasets/navoneel/brain-mri-images-for-brain-tumor-detection/data (Last Accessed: January 10, 2025) https://www.kaggle.com/datasets/sartajbhuvaji/brain-tumor-classification-mri (Last Accessed: January 10, 2025).
